# Synergetic Effects of Granulocyte-Colony Stimulating Factor and Cognitive Training on Spatial Learning and Survival of Newborn Hippocampal Neurons

**DOI:** 10.1371/journal.pone.0005303

**Published:** 2009-04-24

**Authors:** Kai Diederich, Wolf-Rüdiger Schäbitz, Katharina Kuhnert, Nina Hellström, Norbert Sachser, Armin Schneider, Hans-Georg Kuhn, Stefan Knecht

**Affiliations:** 1 Department of Neurology, University of Münster, Münster, Germany; 2 Institute for Neuroscience at the Institute for Clinical Neuroscience, Sahlgrenska Academy, Gothenburg University, Gothenburg, Sweden; 3 Department of Behavioural Biology, University of Munster, Munster, Germany; 4 Sygnis Bioscience GmbH & Co. KG, Heidelberg, Germany; Julius-Maximilians-Universität Würzburg, Germany

## Abstract

Granulocyte-Colony Stimulating Factor (G-CSF) is an endogenous hematopoietic growth factor known for its role in the proliferation and differentiation of cells of the myeloic lineage. Only recently its significance in the CNS has been uncovered. G-CSF attenuates apoptosis and controls proliferation and differentiation of neural stem cells. G-CSF activates upstream kinases of the cAMP response element binding protein (CREB), which is thought to facilitate the survival of neuronal precursors and to recruit new neurons into the dentate gyrus. CREB is also essential for spatial long-term memory formation. To assess the role and the potential of this factor on learning and memory-formation we systemically administered G-CSF in rats engaged in spatial learning in an eight-arm radial maze. G-CSF significantly improved spatial learning and increased in combination with cognitive training the survival of newborn neurons in the hippocampus as measured by bromodeoxyuridine and doublecortin immunohistochemistry. Additionally, G-CSF improved re-acquisition of spatial information after 26 days. These findings support the hypothesis that G-CSF can enhance learning and memory formation. Due to its easy applicability and its history as a well-tolerated hematological drug, the use of G-CSF opens up new neurological treatment opportunities in conditions where learning and memory-formation deficits occur.

## Introduction

Granulocyte-colony stimulating factor (G-CSF), a hematopoietic growth factor known for its prominent role in proliferation and differentiation of hematopoietic cells [1], [2], is one of a surprising variety of peripheral circulating peptides that have the ability to alter CNS functions and structure. Several of these peptides, including G-CSF, have specific receptors in the brain and, most importantly, are even produced in the brain [3]. Recent studies showed that peripheral peptides like erythropoietin [4], Insulin-like growth factor 1 [5], [6], Glucagon-like peptide-1 [7] and ghrelin [8] exert action in the CNS. Some of these factors have been shown to induce neuroplasticity and specifically in the hippocampus, changes in neuronal complexity, neurogenesis and LTP.

The G-CSF ligand and receptor show a broad, predominantly neuronal expression throughout the rat brain, with particularly high expression in the CA3 region of the hippocampal formation and the subgranular zone and hilus of the dentate gyrus [9]. We recently showed that G-CSF attenuates apoptosis and controls proliferation and differentiation of neural stem cells, generally fulfilling the criteria of a classic neurotrophic factor [9]. Moreover, it has been demonstrated that G-CSF after binding to its receptor evokes the MAP kinase pathway by activating ERK 1,2 and 5, upstream kinases of CREB, shown to be essential for spatial long term memory formation [10], [11]. CREB activation is furthermore thought to facilitate the survival of neuronal precursors and to recruit new neurons into the dentate gyrus [12].

The expression pattern of G-CSF ligand and its receptor combined with its strong trophic activity and signalling prerequisitions indicate a prominent role in hippocampal function for G-CSF. We therefore administered G-CSF to rats before and during spatial learning and assessed memory formation and hippocampal neurogenesis.

## Materials and Methods

### Ethics Statement

All behavioral testing was performed during the rats' light cycle between 8:00 a.m. and 1:00 p.m. All experiments were done in accordance with the European Communities Council Directive of 24 November 1986 (86/609/EEC).

### Animals

A total of 60 male Wistar rats (Charles River, Sulzfeld, Germany), weighing 180–200 g upon arrival were used in the experiments. They were housed in groups of two animals in Macrolon cages. All rats were kept under controlled environmental conditions (ambient temperature 22°C, 12-h light/dark cycle, lights on at 7:00 a.m.). Standard laboratory chow (Altromin1324, Lage, Germany) was restricted to 16 g per animal per 24 h. This controlled feeding schedule was continued throughout the whole testing period, keeping the animals' body weight on approximately 85% of the free feeding weight. Tap water was allowed *ad libitum*. The behavioral testing began two weeks after the animals' arrival. Rats were handled daily by the experimenter during this period.

### Apparatus and training

The radial eight-arm maze was constructed of gray plastic with a central platform (35 cm in diameter) and eight arms (each 57.5 cm long and 13 cm wide provided with a 2.0 cm rim) projecting radially from this platform with adjacent arms separated by 45°. Each arm was surrounded by a transparent Plexiglas wall (30 cm high). The maze was elevated 35.0 cm off the floor. A nontransparent food cup (0.5 cm high, 8.5 cm in diameter) which concealed the food reward (Bioserv dustless precision pellets 45 mg, Frenchtown, USA) from view was positioned at the distal end of each arm. The maze was set in an experimental room with several external visual cues. The experimenter monitored the movements of the rats via a video camera mounted above the maze and a TV-screen outside the rats' range of vision. On the first day each rat was allowed to explore the maze. Food pellets were scattered throughout the maze and placed in the food cups. The rat was removed from the maze when all pellets were consumed. The training procedure started on the next day with the food wells of four arms baited (one pellet each) and four arms unbaited. For each rat, the four baited arms were randomly chosen with the restriction that no more than two adjacent arms were baited. The spatial location of the baited arms was constant with respect to extra-maze cues. Each rat was trained with two trials a day. A trial began with the experimenter placing a rat on the central platform of the maze with orientation randomly varying from trial to trial and ended when all reward pellets had been collected. Time between trials for each rat was 90 min. Performance was indexed by the number of errors per trial. Two types of errors were identified: The first entry into an unbaited arm was classified as a reference memory error, which implies that the maximum number of reference memory errors per trial is four. Re-entries into arms visited before were classified as working memory errors. Additionally, we monitored the duration of each trial. Between trials the maze was wiped off with a mild disinfectant. The animals were retested after 26 days for a further three days (2 trials each day). The spatial location of the baited arms for each remained unchanged. To examine a possible treatment effect on locomotor function we divided the total duration of each trial by the amount of arm entries. This value is used as an indicator for locomotor function.

### Experimental design

**Figure 1:**
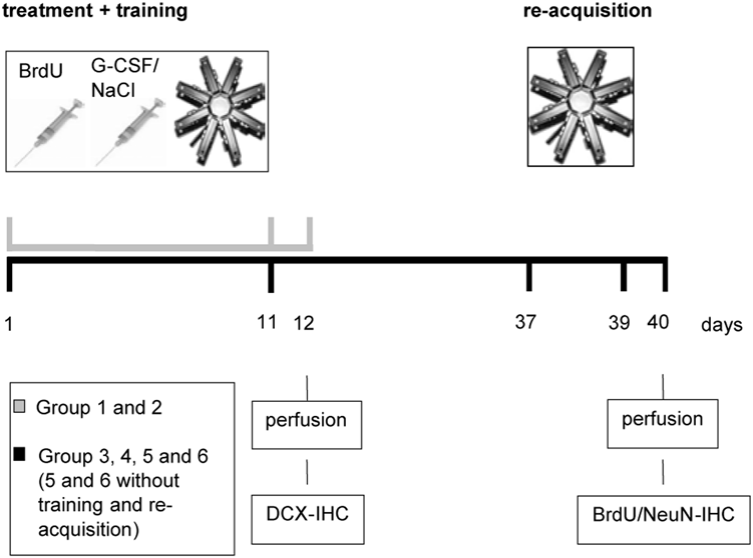
Experimental design showing the different interventions and points of measurement.

The design of the present study is illustrated in Figure 1. The experiments were performed on a total number of 60 animals subdivided into 6 different treatment groups.

The animals of group 1 (n = 10) and 2 (n = 10) received daily injections of G-CSF (group 1) (20 µg/kg, s.c.) or saline (group 2) 1 hour prior to the maze training throughout an 11-day training period in the radial maze. They were subsequently anesthetized and transcardially perfused with 200 ml saline (d12).

The animals of group 3 (n = 10) and 4 (n = 10) also received daily injections of G-CSF (group 3) or saline (group 4) 1 hour prior to the maze training throughout the 11-day training period in the radial maze. The animals were retested after 26 days for further three days (2 trials each day) and were then (d40) anesthetized and transcardially perfused.

Animals of group 5 (n = 10) and 6 (n = 10) were treated daily with G-CSF (group 5) or saline (group 6) over 11 days. Instead of being trained in the radial arm maze, the animals were simply placed on the apparatus every day and were given four food pellets. On day 40 the animals were anesthetized and perfused.

For the purpose of labeling dividing cells each rat received a daily bromodeoxyuridine (BrdU) injection (50 mg/kg/d, i.p.) throughout the 11 day-acquisition-period, 16 hours prior to G-CSF/saline injection.

### Tissue processing

Animals were deeply anaesthetized using a mixture of ketamine (20.38 mg/mL) and xylazine (5.38 mg/mL). Transcardiac perfusion was performed with 0.9% NaCl solution. The brain was removed and postfixed in 4% paraformaldehyde solution for 4 days at 4°C. The tissue was then cryoprotected by 24 h immersion in 30% sucrose-PBS solution. 40-µm sagittal sections were cut on dry ice using a sliding microtome.

### Immunohistostaining

All experiments were done in a fully randomized and blinded fashion. We used two different time points for the detection of neurogenesis in order to differentiate between generation and survival of newborn neurons in the dentate gyrus. For Group 1 (n = 10) and 2 (n = 10) Doublecortin (DCX) was used to determine the amount of newborn neurons within the dentate gyrus after the 11 day-acquisition-period. For Group 3 (n = 10), 4 (n = 10), 5 (n = 10) and 6 (n = 10) BrdU labeling was used for the detection of neurogenesis.

Free-floating sections were treated with 0.6% H_2_O_2_ in Tris-buffered saline (TBS; 0.15 m NaCl, 0.1 m Tris-HCl, pH 7.5) for 30 min. Following extensive washes in TBS, sections were blocked with a solution containing TBS, 0.1% Triton-X100 and 3% normal donkey serum solution for 30 min. The same solution was used during the incubation with antibodies. Primary antibodies were applied overnight at 4°C. For epifluorescence immunodetection, sections were washed extensively and incubated with fluorochrome-conjugated species-specific secondary antibodies. Sections were placed on Superfrost Plus slides (Menzel-Gläser, Germany) and mounted in Prolong Antifade kit (Molecular Probes).

The following antibodies were used: Rat anti-BrdU (1∶500, Accurrate), mouse anti-NeuN (1∶500, Chemicon), goat anti-DCX C-18 (1∶500, Santa Cruz).

### Counting procedures

To determine the number of DCX- or BrdU-positive cells in the hippocampus, every 12th section (480-µm intervals) of one cerebral hemisphere was selected from each animal and processed for immunohistochemistry. All DCX- and BrdU-positive cells in the granule cell layer of the hippocampal dentate gyrus were counted on eight sections per animal. The amount of cells counted was then extrapolated to receive an approximated value for the whole brain. For co-labeling with neuronal marker NeuN to estimate the percentage of neurons among the newly generated cells, 50 randomly selected BrdU-positive cells per animal were analyzed under the confocal microscope. Multiplying the total number of BrdU-positive cells with the percentage of NeuN/BrdU double-positive cells yielded the number of new neurons in the dentate gyrus.

To determine the number of BrdU-positive cells in the olfactory bulb a systematic, random counting procedure, similar to the optical dissector, was used as described by Williams & Rakic (1988) [13]. The volume of each structure was determined by tracing the areas using a semiautomatic stereology system (StereoInvestigator, MicroBrightField, Colchester, VT, USA).

### Statistical analysis

All analyses were performed with the statistical software SPSS (version 13.00 for Windows). One-way analysis of variance (ANOVA) was used to compare data between groups. Two-way ANOVA was used when data of different groups were repeatedly collected over time or under different treatment conditions (e.g. G-CSF/vehicle). For the statistical analysis of radial eight-arm maze performance, two-way repeated measures ANOVA was calculated over 22 trials (acquisition period) and 6 trials (reacquisition period). Post hoc comparisons were made using Fisher protected least significant difference test. A Student's t-test was calculated for the statistical analysis of DCX positive cells. All tests performed were two-tailed and a value of *p*<0.05 was considered to represent a significant difference.

## Results

### Radial maze spatial memory task

**Figure 2:**
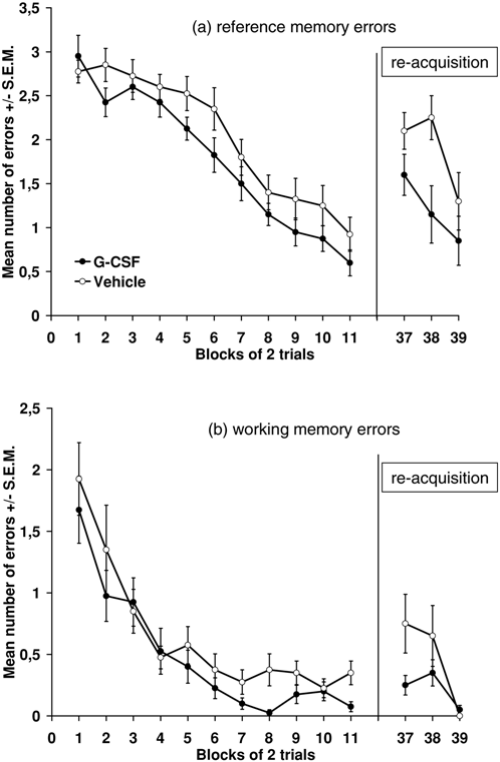
Spatial reference (a) and working (b) memory-formation in rats treated with vehicle or G-CSF during the spatial learning and re-acquisition. G-CSF significantly improved reference memory during the acquisition (Blocks 1–11; p = 0.015; ANOVA with repeated measures) and re-acquisition (Blocks 37–39; p = 0.004) period. Furthermore, G-CSF improves working memory in the re-acquisition (p = 0.033) but not in the initial acquisition phase (p = 0.952).

We examined the effects of G-CSF treatment on reference and working memory during spatial learning. G-CSF treatment significantly reduced the number of reference memory errors (Figure 2A). An ANOVA with repeated measures revealed significant effects of group (F(1,38): 6.552; p = 0.015) and trial (F(1,38): 256.654; p<0.001), but not group by trial interaction (p = 0.325). This effect continued during the reacquisition period 26 days later (ANOVA group (F(1,18): 6,586; p = 0.019), trial (F(1,18): 11.129; p = 0.004), group by trial interaction (p = 0.525)). In order to demonstrate that the differences in the reacquisition trials are not caused by a better acquisition of the task in the first trials we calculated a covariance analysis with the last trial of the acquisition period as a covariate. This analysis revealed a statistically significant group effect between the treatment groups (F(1,17): 5.826; p = 0.027). In contrast to the improvement in the formation of reference memory, G-CSF did not improve working memory to a significant level in the initial acquisition phase (Figure 2A) (ANOVA group p = 0.061, trial (*F*(1,38): 95,019; *p*<0.001), group by trial interaction (p = 0.952)). However, in the reacquisition phase 26 days later G-CSF treated animals made significantly less working memory errors (ANOVA group F(1,18): 5.333 (p = 0.033), trial (F(1,18): 11.155; p = 0.004) group by trial interaction (F(1,18): 5.186, p = 0.035)).

We also examined the effects of G-CSF treatment on locomotor function and appetite, both of which affect performance in the radial arm maze test. There were no differences in locomotor activity and body weight between the G-CSF and saline-treated rats (p>0.05, Student's *t* test, data not shown). Thus, it is unlikely that the differences in maze performance in rats are a secondary effect of motor dysfunction or an altered motivational state.

### Neurogenesis detection

Using DCX immunohistochemistry, we determined the amount of newborn cells after the 11- day-(training)-period. There was no significant treatment effect on the amount of DCX-positive cells (Student's *t* test, p = 0.277; G-CSF-treated animals: 930.6+/−71.6 S.E.M.; vehicle-treated animals: 829+/−55.6).

**Figure 3:**
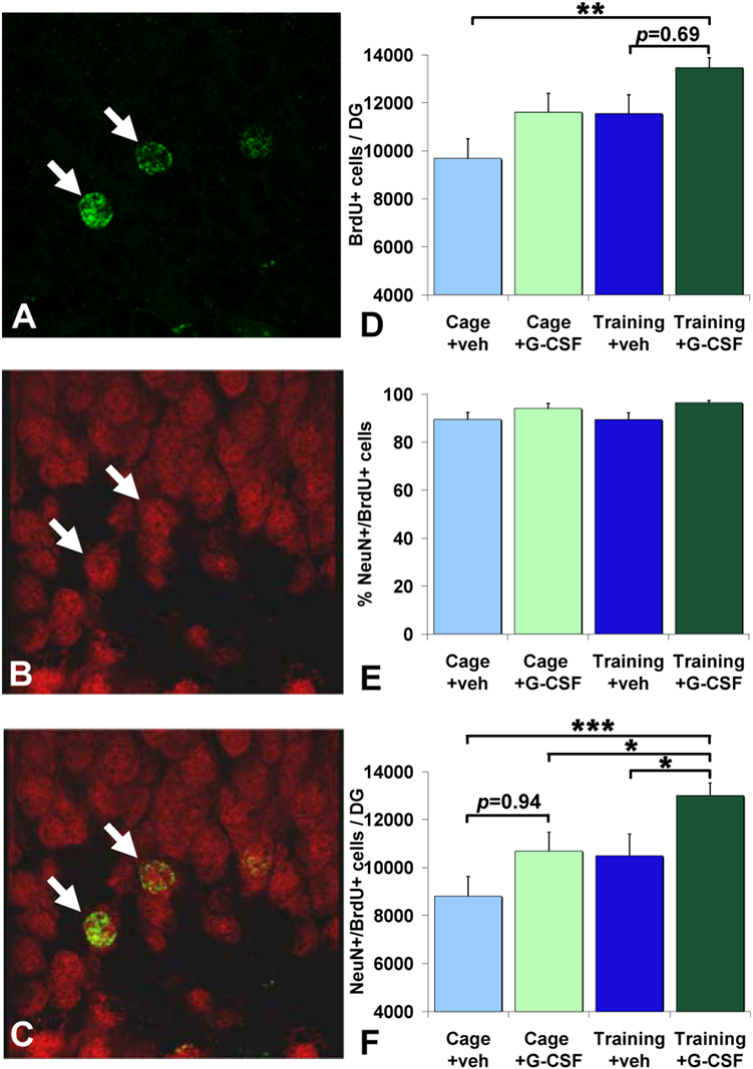
Quantification of neurogenesis by the detection of BrdU/NeuN-expressing cells. A–C: Confocal microscopic images immunohistochemically stained cells. A: BrdU positive cells. B: NeuN positive cells. C: merged image, BrdU/NeuN positive cells (arrows). D–F: Histograms representing the number of (D) BrdU and (F) BrdU/NeuN-expressing cells (mean±S.E.M.) as well as (E) the percentage of BrdU/NeuN positive cells in the dentate gyrus of vehicle and G-CSF treated rats with and without training in the radial-arm maze after the 3-day re-acquisition period. * p<0.05; ** p<0.01; *** p<0.001.

To assess whether G-CSF treatment led to an increased survival of adult-born neurons generated during the 11-day training period, we counted BrdU/NeuN double-positive cells in the dentate gyrus after the reacquisition period (Figure 3). A one way ANOVA revealed significant differences between the four treatment groups in the number of BrdU/NeuN positive cells (F(3,39): 2.356; p = 0.005). Post hoc analyses showed a significant increase in newborn cells in the dentate gyrus of trained/G-CSF-treated animals compared to all other treatment groups. However the most prominent difference was found between trained/G-CSF-treated and non-trained/vehicle-treated animals (p<0.001). G-CSF treatment in combination with the spatial training in the maze increased the amount of BrdU/NeuN double-positive cells by 48%. In untrained, G-CSF treated animals there was an observable increase in BrdU/NeuN positive cells, though not reaching significance when compared to untrained animals treated with the vehicle. (p = 0.09).

A one way ANOVA revealed significant differences between the four treatment groups in the total number of BrdU positive cells (F(3,39): 4.605; p = 0.008). Post hoc analyses showed a significant increase in newborn cells in the dentate gyrus of trained-G-CSF-treated animals compared to non-trained/vehicle-treated animals (p<0.001). Furthermore, there was an observable albeit insignificant difference between the trained-G-CSF-treated group and the trained-vehicle-treated group (p = 0.069). In untrained animals, G-CSF treatment did not result in a statistically significant increase in BrdU positive cells when compared to vehicle treatment (p = 0.094). To investigate possible neuron-specific effects of G-CSF treatment we analyzed the percentage of BrdU/NeuN positive cells in the dentate gyrus. G-CSF treatment exerts no significant neuron-specific effects (one way ANOVA: p = 0.088).

We analyzed the number of BrdU positive cells in the olfactory bulb, another neurogenic niche of the brain. Neither G-CSF treatment alone nor in combination with spatial training led to an increase in the amount of newborn cells in this area (one way ANOVA: p = 0.182; non-trained/vehicle-treated: 56851 cells +/−6098.3 S.E.M.; non-trained/G-CSF-treated: 68428+/−6029.4; trained/vehicle-treated: 54639+/−7696; trained/G-CSF-treated: 75409.9+/−6359).

## Discussion

Our data show that treatment with the hematopoietic factor G-CSF combined with cognitive training improves long-term spatial memory and promotes the survival of newborn hippocampal neurons.

The reduction in reference memory errors during the acquisition period reflects improved acquisition of spatial memory directly after G-CSF treatment. At this stage, G-CSF did not increase the number of newborn neurons measured by DCX-immunohistochemistry. Newly generated neurons in the adult hippocampus must achieve full maturation before they become functional and influence behavior [14]. Therefore, neurogenesis is unlikely to be directly involved in this immediate learning improvement. Learning enhancement by G-CSF at this stage may, however, have been due to an interaction of G-CSF with its receptor in the hippocampus, resulting in CREB activation via MEK/ERK signaling. G-CSF activates the MAP kinase pathway by activating ERK 1,2 and 5 [9]. Recent studies have highlighted the importance of ERK in synaptic plasticity and memory formation across many species, brain areas and types of synapses [15], [16]. CREB is presumed to be a direct downstream target of activated ERK [17]. There is evidence that the activation of CREB in the dorsal hippocampal CA3 region is a critical step in the signaling cascade that leads to the structural changes underlying the formation of long-term memory [18], [19].

In addition to the enhanced performance during spatial learning, G-CSF treatment led to a significant improvement in reference and working memory during reacquisition 26 days later. Spatial learning is initially dependent on the hippocampus, which appears to prepare contents for long-term storage in the neocortex. Newborn neurons in the hippocampus could contribute to learning and memory formation [20]. Furthermore, neuronal turnover may provide plasticity for information storage that more differentiated neurons cannot [21]. Consistent with this idea, the survival of young adult-born neurons can be increased by learning and enriched environments [22]–[25]. In the current study, G-CSF treatment did not increase the number of DCX-positive newborn cells immediately following the 11-day treatment and training period. In contrast, subsequent to the reacquisition, we found an increase in the number of BrdU/NeuN-positive cells in G-CSF treated and trained animals. Adult-born neurons may contribute to cognitive functions if they are functionally integrated in the hippocampal formation. After labeling newly generated hippocampal cells with a retrovirus expressing a reporter gene, van Praag and colleagues [26] showed that, over a period of weeks, new cells developed electrophysiological characteristics very similar to older granule cells. Hence, adult-generated neurons have the ability to become electrophysiologically functional and integrate into the hippocampal formation as granule cells.

There are several studies reporting that learning increases the survival of newborn neurons [22], [24], others which show no effect [27] and still others showing learning may decrease the survival of newborn hippocampal neurons [28]; [29]. Two recently published studies suggest that the age of the labeled cells at the time of learning is critical in determining the effects [30], [31]. In the current study, we used DCX and BrdU as markers for neurogenesis at two time points to evaluate the survival of newborn hippocampal neurons. DCX is a reliable and specific marker that reflects levels of adult neurogenesis and its modulation. The expression of DCX starts as neuroblasts are generated and peaks during the second week [32]. Therefore, this technique is well suited for the detection of immediate effects on neurogenesis. For the detection of longer-term survival of newborn neurons, we injected BrdU on a daily basis throughout the acquisition phase (d1–d11) and quantified BrdU/NeuN-labelled cells following the perfusion of the animals on day 40. In the present study the combination of G-CSF treatment and cognitive training markedly increased the number of BrdU/NeuN-positive cells in the hippocampus, clearly surpassing the survival-enhancing effect of each treatment alone. G-CSF may promote the surviving of newborn cells trough its well-investigated anti-apoptotic effects as well as trough its prominent actions in proliferation and differentiation of neural cells. The mechanism whereby learning increases cell survival has not been fully identified yet. A ‘use it or lose it’ principle is thought to underlie the survival of hippocampal neurons [33]. The finding of the present study suggests that the combination of hippocampus-dependent learning and G-CSF treatment may facilitate the integration of adult-born neurons into existing neural networks and therefore insure their survival.

Numerous reports have described the efficacy of G-CSF in animal models of different neurological diseases including stroke [9], [34]–[37], Parkinson's disease [38], [39], and recently Alzheimer's disease [40] and amyotrophic lateral sclerosis [41]. Interestingly, in animal models of stroke and Alzheimer's disease, treatment with G-CSF has been shown to ameliorate cognitive deficits [40], [42]. Although, recent studies have begun to explore G-CSF-related mechanisms of action in various disease models, little is known about its function in the healthy brain. A more detailed understanding of the physiological role of G-CSF in the healthy brain may, however, open new insights into disease relevant mechanisms. We therefore investigated the effect of peripheral administered G-CSF on learning and memory formation and the generation and survival of newborn hippocampal neurons in healthy rats.

Overall, the findings from the present study support the hypothesis that G-CSF can enhance learning and memory formation. Most importantly because of its easy applicability and its history as a well-tolerated hematological drug, learning enhancement by G-CSF opens up new neurological treatment opportunities in conditions where learning and memory-formation deficits occur.
